# Metatranscriptomic Sequencing Suggests the Presence of Novel RNA Viruses in Rice Transmitted by Brown Planthopper

**DOI:** 10.3390/v13122464

**Published:** 2021-12-09

**Authors:** Shufen Chao, Haoran Wang, Qing Yan, Long Chen, Guoqing Chen, Yihong Wu, Baozhong Meng, Lixin Jin, Xudong Zhu, Guozhong Feng

**Affiliations:** 1 State Key Laboratory of Rice Biology, China National Rice Research Institute, Hangzhou 311400, China; chaoshufen@163.com (S.C.); wanghaoran.12@foxmail.com (H.W.); qyan2005@hotmail.com (Q.Y.); chenlong@caas.cn (L.C.); chenguoqing@caas.cn (G.C.); redone233@163.com (Y.W.); zhuxudong@caas.cn (X.Z.); 2 Department of Molecular and Cellular Biology, University of Guelph, Guelph, ON N1G 2W1, Canada; bmeng@uoguelph.ca; 3 Fuyang Plant Protectin Station, Hangzhou 311400, China; kinlixin@163.com

**Keywords:** rice, brown planthopper, metatranscriptomics, small RNA, RNA viruses

## Abstract

Viral pathogens are a major threat to stable crop production. Using a backcross strategy, we find that integrating a dominant brown planthopper (BPH) resistance gene *Bph3* into a high-yield and BPH-susceptible *indica* rice variety significantly enhances BPH resistance. However, when *Bph3*-carrying backcross lines are infested with BPH, these BPH-resistant lines exhibit sterile characteristics, displaying panicle enclosure and failure of seed production at their mature stage. As we suspected, BPH-mediated viral infections could cause the observed sterile symptoms, and we characterized rice-infecting viruses using deep metatranscriptomic sequencing. Our analyses revealed eight novel virus species and five known viruses, including a highly divergent virus clustered within a currently unclassified family. Additionally, we characterized rice plant antiviral responses using small RNA sequencing. The results revealed abundant virus-derived small interfering RNAs in sterile rice plants, providing evidence for Dicer-like and Argonaute-mediated immune responses in rice plants. Together, our results provide insights into the diversity of viruses in rice plants, and our findings suggest that multiple virus infections occur in rice plants.

## 1. Introduction

Rice (*Oryza sativa*) is one of the most important global cereal crops and feeds roughly half of the world’s population. Rice is cultivated in hot and humid climates, an environment also suitable for the propagation of viruses. The first major outbreak of rice viruses was caused by the *Rice dwarf virus* (RDV) in Japan in 1897 [1]. To date, only 22 rice viruses have been documented [1,2,3]. These viruses result in diseases that pose serious threats to stable rice production. Virus-infected rice plants can show various symptoms, including pronounced stunting, darker green leaves, increased tillering, and elongation failure. Most rice viruses are transmitted by rice insect pests, including the planthopper and leafhopper [1,4]. The brown planthopper (*Nilaparvata lugens* Stål, BPH) is the most destructive pest of rice and a substantial threat to rice production, causing billions of dollars in crop losses annually [5,6]. Developing BPH-resistant rice cultivars is considered the most economically effective and environmentally friendly intervention to control this insect pest. In this work, validation of BPH resistance in the backcrossed first generation (BC_1_F) was completed by selecting and cultivating Rby1 and Rby2 carrying *Bph3*. Both of these rice lines grow as healthy rice plants following BPH infestation, but become sterile at a mature stage. We speculated that the observed sterile symptoms in BC_1_F are due to BPH-transmitted viruses and subsequent viral infections. To test this hypothesis, we first tried to isolate the virus from BPH-infested sterile rice plants. However, we did not find any virus. Then, we used deep metatranscriptomic sequencing to detect viral genomes in the sterile rice plants, and virus-derived small interfering RNAs (vsiRNAs) were also identified through small RNA sequencing. Our analyses reveal multiple known and many novel RNA viruses as well as abundant vsiRNAs. Together, the results provide insights into the diversity of viruses in rice plants, and our findings indicate that multiple virus infections occur in sterile rice plants.

## 2. Materials and Methods

### 2.1. Plant Materials and Sample Collection

The rice cultivar Ms55 and TZ21 used in this work were obtained from the Chinese Rice Germplasm Resources Centre (Hangzhou, China). Ms55, a high-yield cultured elite *indica* variety, was used as recurrent parent. TZ21 harboring resistance gene *Bph3* was used as donor parent [5]. Two *Bph3*-carrying lines, Rby1 and Rby2, were selected from BC_1_F population by backcrossing between recurrent parent Ms55 and donor TZ21. Rice plants from lines Rby1 and Rby2 were grown in greenhouse at the China National Rice Research Institute, Hangzhou, China. Leaf and stem samples of rice plants Rby1-21 from Rby1 and Rby2-45 from Rby2 were harvested and placed on dry ice until they were moved to the laboratory, where the samples were stored at −80 °C prior to RNA isolation.

### 2.2. Brown Planthopper Maintenance

Brown planthopper (BPH) was collected from a rice field at China National Rice Research Institute, Hangzhou (where the BPHs are mixed biotypes and mainly biotype 2) and maintained on the susceptible cultivar Ms55 under greenhouse conditions at China National Rice Research Institute.

### 2.3. Evaluation of Brown Planthopper Resistance

Bioassays were conducted following the method described by Liu with minor modification [5]. Line seeds were germinated in petri dishes, and 30 seeds from each plant were sown in a 10 cm diameter plastic pot with a hole at the bottom. Seven days after sowing, seedlings were thinned to 20 plants per pot. At the second-leaf stage, the seedlings were infested with second- and third- instar BPH nymphs at 10 insects collected from rice fields per seedling. When all the Ms55 plants were dead, the seedling mortality of other cultivars or lines was recorded. Three replicates were used for each cultivar or line.

### 2.4. Metatranscriptomic Sequencing

#### 2.4.1. Sample Processing and Sequencing

Total RNA were isolated from rice samples Rby1-21 and Rby2-45. To isolate total RNA, the samples were homogenized in 700 μL of lysis buffer by TissueRuptor (Qiagen, Hilden, Germany). Total RNA was then extracted by using an RNeasy Plus mini kit according to the manufacture’s protocol (Qiagen). The quality of the resultant RNA was evaluated using an Agilent 2100 bioanalyzer (Agilent Technologies, Palo Alto, CA, USA) before library construction. rRNA was removed by using a Ribo-Zero Magnetic kit (Plant Leaf) (Illumina, San Diego, CA, USA), the remaining RNA was fragmented, reverse-transcribed into cDNA, ends repaired and adaptor ligated by using an Illumina Truseq Total RNA Library Preparation Kit. Finally, ligated RNA was amplified to generate a cDNA library. Paired-end (150 bp) sequencing of each library was then performed on the HiSeq 2500 platform (Illumina). Library preparation and sequencing were carried out by Novogene Co., Ltd. (Tianjin, China). 

#### 2.4.2. Sequence Read Assembly and Virus Discovery

Sequencing reads were assembled de novo by using Trinity. The assembled contigs were first compared against the database of all reference RNA virus proteins downloaded from Genbank by using BLASTX with an E value cutoff at 1E-5 to maximize sensitivity while minimizing false-positive results. The resultant contigs were then compared to a nonredundant nucleotide (nt) and protein (nr) database to remove non-viral sequences. We also performed domain-based BLAST to detect highly divergent viruses. The assembled contigs were compared to the Conserved Domain Database (CDD) version 3.16 with an expected value threshold of 1E-2. The quality-filter virus contigs with unassembled overlaps were then merged using SeqMan implemented in the Lasergene software package v7.1 (DNAStar). To confirm the assembly results, reads were mapped back to the virus genomes with Bowtie2 and inspected using an integrated genomics viewer (IGV) for any assembly errors. The final sequences of the virus genomes were obtained from the majority consensus of the mapping assembly. Samtools were used to determine the sequencing depth and coverage [7].

#### 2.4.3. Virus Genome Annotation

The potential ORFs of the newly identified virus genomes were annotated based on predicted amino acid sequences and conserved positions in the genome compared to the closest related virus genome available in GenBank. Functional domains within each ORF were identified using BLAST against the Conserved Domain Database (CDD) with an expected value threshold of 1E-5. 

For viruses with multiple RNA segments, we used various strategies to search for viral genome segments, as described previously [8]. Non-RdRp segments were identified by homology to the proteins of related reference viruses. Other potential segments that had no homology to sequences in the database were identified by using an in silico approach that utilizes information on RNA quantity, protein structure, and/or conserved genome termini. To determine which segments belong to the same virus, we checked (1) the sequencing depth of the segments; (2) the presence of conserved regulatory sequences in the non-coding regions located at termini of the viral genome; and (3) the phylogenetic positions of related viral proteins.

We used RT-PCR and Sanger sequencing to verify the presence of each putative virus in the samples. Primers were designed from the contigs assembled from next-generation sequencing. Genome walking and RT-PCR were used to fill in sequence gaps for putative viral contigs that contained incomplete sequences. To exclude the possibility that putative viral contigs expressed endogenous virus elements (EVEs), DNA isolated from the corresponding samples was examined by PCR and Sanger sequencing. Genome termini were determined using 5′/3′ RACE kits (TaKaRa).

#### 2.4.4. Quantification of Relative Transcript Abundances

To determine the abundance of RNA transcripts, we mapped total reads to the assembled genes or genomes using Bowtie [9]. The reads were analyzed with RSEM [10]. The relative abundance of each transcript is presented as transcripts per million (TPM).

### 2.5. Phylogenetic Analysis

To determine the phylogenetic relationships of the newly identified RNA viruses, the amino acid sequences of the viral RNA-dependent RNA polymerase (RdRp) [11] of these viruses identified in this study and those retrieved from GenBank were aligned to infer their evolutionary relationship. The viral RdRp sequences were then aligned using the E-INS-I algorithm in MAFFT (version 7.429) [12]. Molecular phylogenetic trees were constructed using the neighbor-joining method in MEGA (version 7.0) using bootstrap tests with 1000 replicates [13]. The generated phylogenetic tree was used to infer the relationship between the virus sequences identified in our study and other published viral sequences from the corresponding viral genera.

### 2.6. Small RNA Library Construction and Sequencing

One microgram of total RNA isolated from each rice sample was used to generate a library of small RNAs using a TruSeq Small RNA Sample Preparation Kit (Illumina) according to the sample-preparation instructions. Briefly, small RNA (<40 nt) was ligated with a single-stranded 3′-adapter and a bar-coded 5′-adapter. Ligated small RNA was reverse transcribed and amplified by PCR to generate individual DNA colony template library. Both libraries of Rby1-21 and Rby2-45 were used for 50 bp single-end sequencing by the Illumina HiSeq 2000 platform in two lanes.

### 2.7. Small RNA Analysis

Bioinformatic analysis of small RNA data was performed using the CLC Genomic Workbench software package (Qiagen). Briefly, small RNA reads were quality-checked; low-quality reads and adapter sequences were first removed from the raw small RNA data set. Trimmed small RNA sequences shorter than 15 nucleotides were discarded. The remaining reads were mapped to the rice genome to remove host-related reads. The unmapped reads were subsequently mapped to putative viruses with the same stringency settings.

## 3. Results

### 3.1. Bph3-Carrying Backcross Rice Lines Infested by BPH Are Sterile

**Figure 1:**
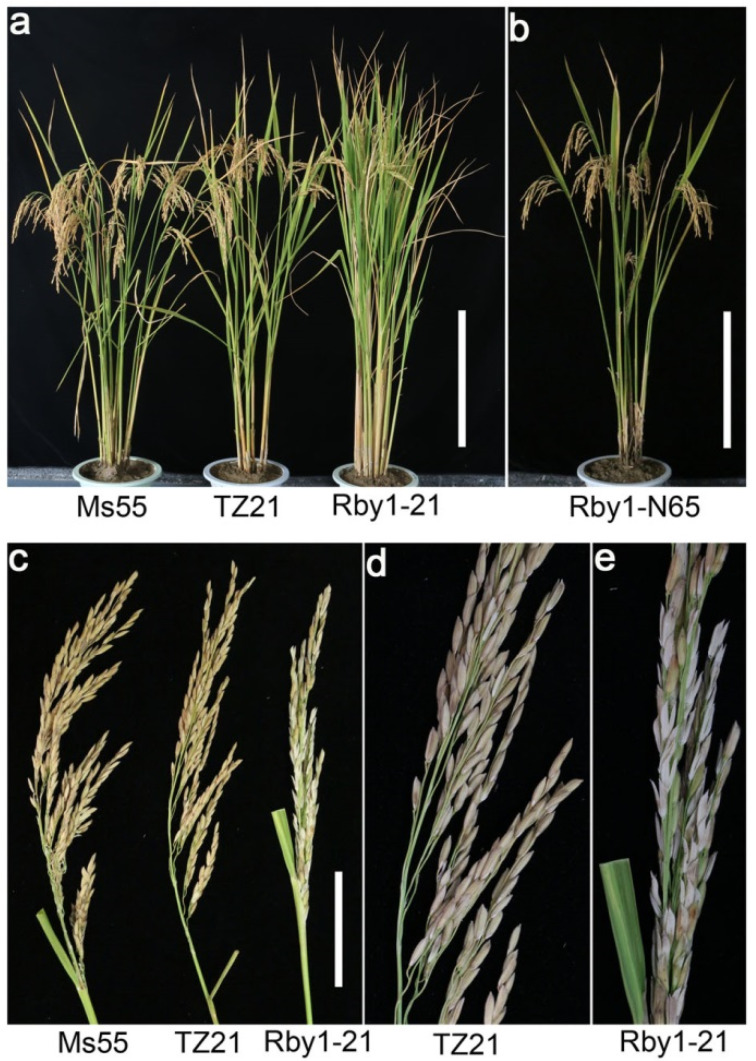
Phenotypic analysis of first-generation rice backcrosses. (**a**) Mature Ms55 (**left**), TZ21 (**middle**), and Rby1-21 (**right**). Rby1-21 is shown as representative rice plants from lines Rby1 and Rby2 infested by BPH, rice plants were grown in soil for 18 weeks. Bar = 30 cm; (**b**) Mature Rby1-N65. Rby1-N65 is shown as a representative rice plant from line Rby1 or Rby2 not infested with BPH. Bar = 30 cm; (**c**) mature panicles of TZ21 (**left**), Ms55 (**middle**), and Rby1-21 (**right**). Bar = 10 cm; (**d**) large images of TZ21 panicle; (**e**) large image of Rby1-21 panicle.

To develop an *indica* rice variety resistant to BPH, Ms55 was used as a recurrent parent to backcross with the TZ21 line that harbors a BPH resistance gene, *Bph3*. These crosses resulted in BC_1_F population that may be resistant to BPH (Figure 1a and Figure S1). Two *Bph3*-carrying *indica* backcross lines, Rby1 and Rby2, were selected from the BC_1_F population. To test whether *Bph3* gene integration can improve BPH resistance, Rby1 and Rby2 rice plants were grown under typical greenhouse conditions, and starting at the seedling stage, each line was infested with BPH collected from rice fields in Hangzhou [6]. Following BPH infestation we found *Bph3*-carrying Rby1, Rby2, and TZ21 lines were not visibly damaged, while 100% of Ms55 plants were dead at 19 days (Figure S2). Using a fixed number of BPH, we found the BPH population steadily increased over time on Ms55, while in the first few hours of introduction, the BPH population dramatically decreased on Rby1, Rby2, and TZ21. These observations suggest Rby1 and Rby2 have a strong resistance to BPH. Interestingly, while BPH-infested Rby1 and Rby2 lines display similar vegetative growth as Ms55 or TZ21, both lines had panicle enclosure. This panicle enclosure resulted in few seeds and is a characteristic of sterile rice plants (Figure 1c–e). Importantly, Rby1 or Rby2 plants not infested with BPH were fertile and displayed similar agronomic traits as Ms55 or TZ21, including earing and seed formation (Figure 1b). 

### 3.2. Identification of Multiple RNA Viruses in BPH-Induced Sterile Rice Plants Using Deep Metatranscriptomic Sequencing

#### 3.2.1. Deep Metatranscriptomic Sequencing of BPH-Induced Sterile Rice Plants

Rby1 or Rby2 plants not infested with BPH grew as healthy plants (Figure 1b) and only displayed sterile symptoms when infested with BPH. Interestingly, these sterile rice plants did not exhibit disease symptoms indicative of fungal or bacterial infections. As BPH infestation resulted in sterility and BPH is known to transmit viruses to rice plants, we speculated BPH-derived rice viruses might be causing sterility in the Rby1 and Rby2 rice lines. To test our hypothesis that BPH-derived viruses result in Rby1/Rby2 rice sterility, we performed deep metatranscriptomic sequencing of rice plants following BPH infestation. Specifically, we characterized the Rby1-21 Rby1 line and the Rby2-45 Rby2 line. RNA sequencing of rRNA-depleted libraries yielded 70,923,042 reads, 12.69 GB of data for Rby1-21, and 84,627,146 reads, 10.64 GB of data for Rby2-45. De novo assembly resulted in 328,146 contigs for Rby1-21 and 586,111 contigs for Rby2-45 (Table S1).

#### 3.2.2. Identification of Previously Known Viruses

Using our assembled contigs, we identified five previously known viruses in Rby1-21 and Rby2-45. Rice Tombus-like virus 1 (RTV1) and rice ragged stunt virus (RRSV) were present in both Rby1-21 and Rby2-45, while Rice Picorna-like virus 1 (RPiV1), Rice Toti-like virus (RtoV), and a BPH virus, Nilaparvata lugens reovirus (NLRV), were present only in Rby2-45 (Table S1). We also used RT-PCR to verify presence of these known viral sequences. 

#### 3.2.3. Identification of Novel Viruses

**Figure 2:**
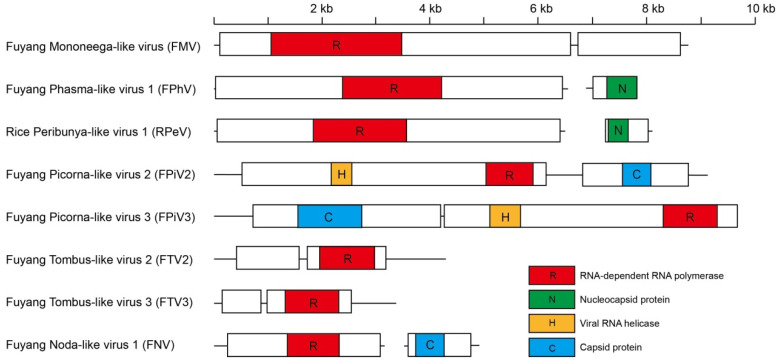
Genome structures of novel viruses. Predicted viral proteins homologous to known viral proteins are shown according to their putative functions.

Using a BLAST-based method for viral genomes in GenBank, we identified eight novel viruses in Rby1-21 and Rby2-45, including three negative-sense RNA viruses and five positive-sense RNA viruses (Figure 2 and Table 1). Fuyang Mononega-like virus (FMV) and Rice Peribunya-like virus (RpeV) were present in both Rby1-21 and Rby2-45 (Table 1). Fuyang Picorna-like virus 2 (FpiV2) and Fuyang Noda-like virus (FNV) were only present in Rby1-21 (Table 1). Fuyang Phasma-like virus (FPhV), Fuyang Tombus-like virus 2 (FTV2), Fuyang Tombus-like virus 3 (FTV3), and Fuyang Picorna-like virus 3 (FpiV3) were only present in Rby2-45 (Table 1). Taken together, we identified four novel RNA viruses in Rby1-21 and six novel RNA viruses in Rby2-45. We also used RT-PCR to verify presence of these novel viruses.

**Figure 3:**
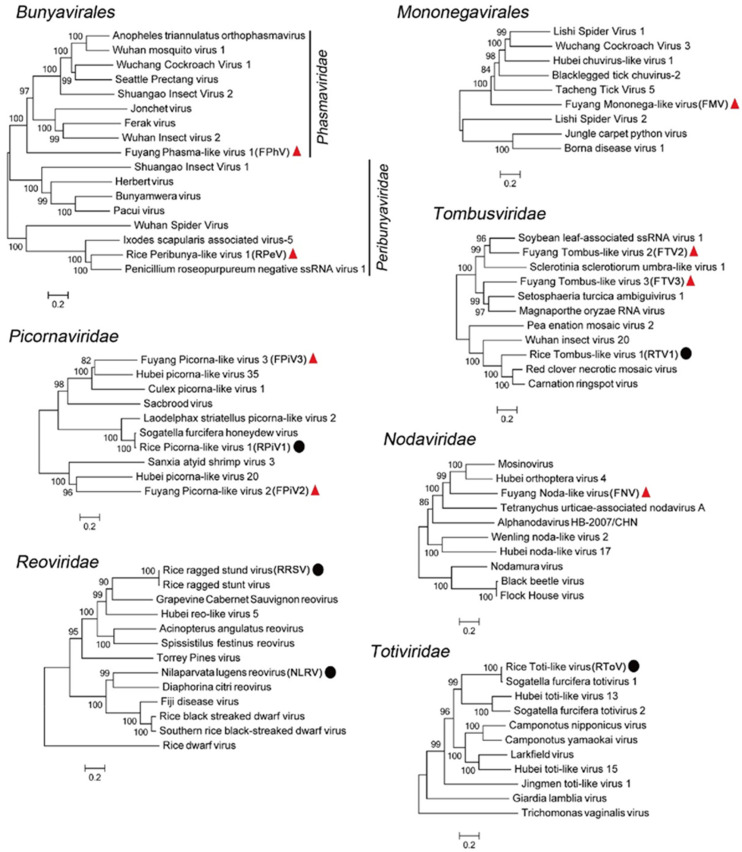
Evolutionary relationship of identified viruses in rice plants Rby1-21 and Rby2-45. The maximum likelihood phylogenetic trees show the position of novel viruses (solid red triangles) and known viruses (solid black circles) in the context of their closest relatives.

We identified three novel negative-sense RNA viruses, all of which were classified within the *Mononegavirales* and *Bunyavirales* orders (family of *Phasmaviridae* and *Peribunyaviridae*) (Table 1). For all these novel negative-sense RNA viruses, the predicted RpeV amino acid sequences were most similar (48% identity) to the Penicillium roseopurpureum negative ssRNA virus 1 sequence (MG887749). The predicted amino acid sequences of the FPhV polyprotein were 19% identical to the Shuangao Insect Virus 1 sequence (NC_031221). In addition, the predicted amino acid sequences of FMV ORF1 contain an RdRp domain similar to Tacheng Tick Virus 5 (NC_028264), which has a 33% identity to FMV (Table 1 and Figure 2). In the RdRp phylogeny, RpeV clustered within the family *Peribunyaviridae*, FPhV belonged to the family *Phasmaviridae*, both of which belong to the order *Bunyavirales* (Figure 3). However, FMV was grouped within a currently unclassified family in the *Mononegavirales* order (Figure 3 and Figure S3).

We discovered five novel positive-sense RNA viruses (Table 1) classified within the *Tombusviridae, Picornaviridae*, and *Nodaviridae* families (Figure 3). Two positive-sense RNA viruses, FTV2 and FTV3, belong to the *Tombusviridae* family within the RdRp phylogeny (Figure 3). The predicted amino acid sequences of FTV2 ORF2 were 56% identical to Soybean leaf-associated ssRNA virus 1 (KT598231). The predicted amino acid sequences of FTV3 ORF2 were 42% identical to Setosphaeria turcica ambiguivirus 1 (MK279508) (Table 1). The remaining positive-sense RNA viruses, FpiV2 and FpiV3, were grouped within *Picornaviridae* (Figure 3). The predicted amino acid sequences of FpiV2 ORF1 contained an RdRp domain that is 46% identical to the corresponding region of Hubei picorna-like virus 20 (NC_033000). The predicted amino acid sequences of FpiV3 ORF2 were 57% identical to Hubei picorna-like virus 35 (NC_033195) (Table 1). FNV clustered within the *Nodaviridae* family (Figure 3) and the predicted amino acid sequence of FNV RNA1 were 32% identical to Hubei orthoptera virus 4 (NC_033311) (Table 1).

### 3.3. Small RNA Analysis Suggests Active Virus Infections in BPH-Infested Sterile Rice Plants

To exclude the possibility that our phylogenetic results represent rice Endogenous Viral Element (EVE) sequences or surface contamination, we mapped raw reads from Rby1-21 and Rby2-45 genomes to our set of BLAST-derived candidate viruses [14]. We found no significant reads mapping to the genome, suggesting no genomic copies of these viruses.

Rice plants utilize small RNA pathways for viral defense [15]. When viruses are actively infecting a rice plant, there is often the presence of antiviral immune responses. From this, we quantified vsiRNAs in infected rice plants as a surrogate for active viral infection. We constructed small RNA libraries from BPH-infested Rby1-21 and Rby2-45 lines. Single-end 50 bp sequencing of both libraries resulted in 24,822,021 and 23,341,707 reads for Rby1-21 and Rby2-45, respectively. 

To map these resulting small RNA reads to putative viruses, we first removed rice-specific small RNA reads and then aligned the remaining reads to viral genomes (Table S1). Small RNAs represented by less than 200 reads were excluded due to the likelihood of random degradation. Our alignment results suggested abundant small RNAs mapped to putative viruses (Table S2). Abundant small RNAs in Rby1-21 were identified for RTV1 (134,715 reads) and RRSV (7108 reads). Abundant small RNAs in Rby2-45 were identified for RTV1 (109,437 reads)**,** RRSV (23,558 reads) and RpeV (3639 reads). 

**Figure 4:**
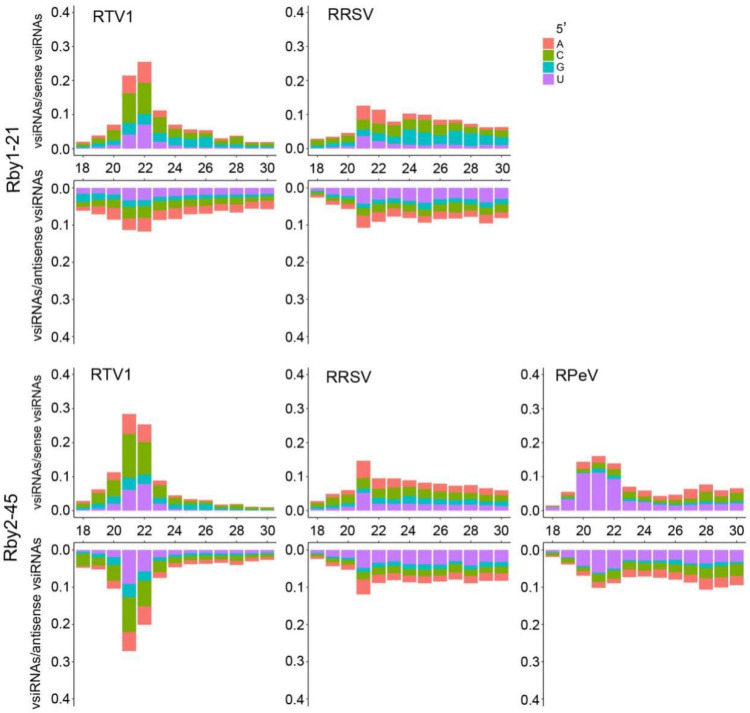
Small RNA analysis of RTV1, RRSV, and RpeV in Rby1-21 and Rby2-45. Size distribution (18 to 30 nt) and strand directionality of virus-derived small RNA arising from RTV1 and RRSV from Rby1-21 as well as RTV1, RRSV and RpeV from Rby2-45. Relative abundance of differing-size sense vsiRNAs (**top**) is shown as the proportion of sense vsiRNAs. Relative abundance of differing-size antisense vsiRNAs (**bottom**) is shown as the proportion of antisense vsiRNAs. Bars plotted above the *x* axis represent vsiRNAs mapping to the positive strand. Bars plotted below the *x* axis represent vsiRNAs mapping to the negative strand. Bars are colored according to the proportions of vsiRNAs starting with A, C, G, and U.

To test whether the presence of these small RNA sequences was due to active virus infections, we next analyzed all 18–30 nt small RNAs from the identified viruses in BPH-infested Rby1-21 and Rby2-45. VsiRNAs detected in plants infected with RNA viruses are typically 21 nucleotides long and produced by Dicer-like 4 (DCL4), whereas 22-nucleotide vsiRNAs are produced by Dicer-like 2 (DCL2) [16,17]. We observed small RNA reads within a size distribution of 21 to 22 nt for RTV1 and RRSV in Rby1-21 as well as RpeV, RTV1, and RRSV in Rby2-45. These small RNAs occurred in both sense and antisense orientations (Figure 4), suggesting that vsiRNAs were produced from double-stranded RNA replicative intermediates and that a rice Dicer-like enzyme cleaved double-stranded RNAs into vsiRNA. A total of 21-nucleotide vsiRNAs occurred in any region of identified genomic RNAs (Figure S4), and those derived from antisense showed a high proportion for each virus (Table 2). However, small RNAs mapping to other putative viruses were not abundant (<200 small RNA reads). This included four viruses in Rby1-21 and eight viruses in Rby2-45. Collectively, our data provide evidence for a Dicer-like and Argonaute-mediated immune response in rice plants. 

## 4. Discussion

Viruses are a major threat to global rice production [18]. Here, we use deep transcriptomic sequencing to describe a diverse set of new viruses in Rby1-21 and Rby2-45 rice lines. We identified 13 complete or nearly complete viral genome sequences, 8 of which are novel and previously undescribed, including 3 negative-sense RNA viruses and 5 positive-sense RNA viruses. We also identified five known viruses containing two positive-strand viruses, RTV1 and RPiV1, as well as three double-stranded viruses, RRSV, NLRV, and RToV [1,19,20]. It is worth noting that RPiV1 and RToV were first discovered in the whitebacked planthopper, *Sogatella furcifera* [20,21]. Here, we find that both RPiV1 and RToV viruses have a high abundance in Rby2-45, suggesting rice plants are their hosts.

Traditionally, virus discovery has focused on viruses that are pathogenic to their hosts and can be isolated. These viruses cause severe diseases and induce obvious symptoms in their plant or animal hosts. However, viral infections can also be asymptomatic or induce inconspicuous symptoms in their hosts. These viruses may accumulate in relatively low titers in their host organisms or become latent such that virus production ceases. Consequently, these mild viruses are not easily isolated or cultured from their hosts using traditional methods. Metatranscriptomics has emerged as a powerful approach to uncover hidden viruses in many organisms, including humans, arthropods, and plants [8,11,22,23,24,25,26,27]. Metatranscriptomics provides sufficient coverage to reconstruct complete viral genomes and allows a relatively straightforward characterization of viral diversity [8]. In this report, we used metatranscriptomics to detect multiple viruses in the sterile samples. To the best of our knowledge, this is the first comprehensive high-throughput survey of viral sequences associated with rice plants.

RNA interference (RNAi) in plants can function as an antiviral defense mechanism against invading viruses. To counteract RNA silencing, many plant viruses have evolved viral suppressors of RNA silencing (VSR) that target various components of the plant RNAi machinery. Different plants show different symptoms and produce different vsiRNA profiles, likely due to differences in plant RNAi components [28]. vsiRNA originating from highly structured sense viral RNA was first identified in *Cymbidium ringspot virus* (CymRSV), a member of the family *Tombusviridae* [29]. In our study, RTV1 was grouped within the *Tombusviridae* family and had a strong sense strand bias in Rby1-21 and Rby2-45. RRSV is a member of the *Reoviridae* family and contains a double-stranded RNA genome. RRSV vsiRNAs also showed a strong sense strand bias in Rby1-21 and Rby2-45, suggesting RRSV-derived vsiRNA originated from highly structured single-stranded viral RNA. Similar results were observed in singly (RRSV) or doubly (RRSV and SRBSDV) virus-infected rice plants [30]. Taken together, our results strongly suggest rice-plant-encoded, DICER-like enzymes recognize highly structured regions RTV1 and RRSV viral ssRNAs and process them into siRNAs.

Breeding pest-resistant rice cultivars is an important approach for maintaining crop yields [31,32,33]. Here, we backcross Ms55, a BPH-sensitive *indica* rice variety, with the *Bph3*-gene containing TZ21 line to produce a BPH-resistant BC_1_F line. Interestingly, we determined that BC_1_F lines are sterile only after BPH infestation. Using deep metatranscriptomics sequencing and vsiRNA analysis, we determined this sterility is likely due to infection by BPH-derived viruses. Our data suggest that the BPH-infested Rby1-21 line harbor active RTV1 and RRSV viral infections. Similarly, we find that the BPH-infested Rby2-45 line harbors active RTV1, RRSV, and RPeV viral infections. Notably, our sequencing failed to identify DNA polymerase sequences indicative of DNA viruses. We identified numerous RNA virus sequences that lacked sequence similarity to rice reference sequences. Interestingly, these RNA virus sequences displayed signatures of DCL2, DCL3, or DCL4 processing (high levels of 21–24 nt vsiRNAs), suggesting they may also be of a viral origin [34,35]. BPH is known to harbor viruses and is capable of transmitting these viruses to rice plants. Our work here suggests that the observed sterile traits of BPH-infested BC_1_F rice may be caused by BPH-transmitted RNA viruses. Further studies are required to test this specific hypothesis. Additionally, our results showed that rice plants did not display obvious disease symptoms when they were co-infected by multiple viruses. Viruses in mixed infections can interact with each other in different ways [36,37]. In this study, we identified 6 viruses in Rby1-21 and 11 viruses in Rby2-45, respectively. We speculate that antagonistic interaction of coexisting viruses may occur in the sterile plants. Further experiments are necessary to prove the relationship between disease symptoms and co-infections of viruses. In conclusion, our findings provide valuable new information about the viruses that rice pests carry and have significant implications for global rice production.

## Figures and Tables

**Table 1:** Classifications, genome characteristics, and abundance of the novel viruses.

Virus Name	Classification	Genome Size (bp)	Abundance Estimation (TPM)	Rice Samples	Closest Relative (RdRp aa Identity)
Negative-sense RNA viruses					
FMV	Mononegavirales	8922	64.96 21.24	Rby1-21 Rby2-45	Tacheng Tick Virus 5 (33%)
FPhV	Phasmaviridae	6709	12.19	Rby2-45	Shuangao Insect Virus 1 (19%)
RpeV	Peribunyaviridae	6549	13.29 721.39	Rby1-21 Rby2-45	Penicillium roseopurpureum negative ssRNA virus 1 (48%)
Positive-sense RNA viruses					
FpiV2	Picornaviridae	9287	17.12	Rby1-21	Hubei picorna-like virus 20 (46%)
FTV2	Tombusviridae	4392	11.14	Rby2-45	Soybean leaf-associated ssRNA virus 1 (56%)
FpiV3	Picornaviridae	9716	43.39	Rby2-45	Hubei picorna-like virus 35 (57%)
FTV3	Tombusviridae	3660	22.33	Rby2-45	Setosphaeria turcica ambiguivirus 1 (42%)
FNV	Nodaviridae	3156	49.75	Rby1-21	Hubei orthoptera virus 4 (32%)

**Table 2:** Abundance and distribution of small interfering RNA in rice plants.

Viruses (Ricae Samples)	siRNA		
	Total	Sense	Antisense
RTV1 (Rby1-21)	134,715	13,456 (9.09%)	121,609 (90.09%)
RRSV (Rby1-21)	7108	1866 (26.25%)	5242 (73.75%)
RTV1 (Rby2-45)	109,437	39,624 (36.21%)	69,813 (63.79%)
RRSV (Rby2-45)	23,558	7492 (31.80%)	16,066 (68.20%)
RpeV (Rby2-45)	3639	1065 (29.27%)	2574 (70.73%)

## Data Availability

Small RNA reads have been deposited in the NCBI Sequence Read Archive (SRA) under accession number SRR16887719 (BioProject accession number PRJNA778875). All viral genome sequences generated in this study have been deposited in the GenBank database under accession numbers (MT317153-MT317177), and raw sequence data reads are available at the NCBI SRA under accession number SRR11300915 and SRR11300916 (BioProject accession number PRJNA612173).

## Supplementary Materials

The following are available online at https://www.mdpi.com/article/10.3390/v13122464/s1, Table S1: Data generated in this study and summary of the viruses; Table S2: Number of small RNA reads mapped to viruses; Figure S1: Scheme of backcross breeding used in the current study; Figure S2: Seedling mortality rate of Ms55, Rby1, and Rby2 infested with BPH; Figure S3: The unrooted ML phylogeny of the novel FMV; Figure S4: Highly abundance 21-nt vsiRNAs to target the viral genomes.
