# Supplementary material for: Metatranscriptomic Sequencing Suggests the Presence of Novel RNA Viruses in Rice Transmitted by Brown Planthopper
Source: Viruses. 2021 Dec 9;13(12):2464. doi: 10.3390/v13122464 (PMC8708968; doi:10.3390/v13122464)
Supplement: Supplementary file 1 [file viruses-13-02464-s001.zip › viruses-1492439-supplementary.pdf]

Supplementary Tables

Table S1. Data generated in this study and summary of the viruses

Rice samples	Data generated			viruses	
	No. of reads	data yield (Gb)	contigs	Novel viruses	Known viruses
Rby1-21	70,923,042	12.69	328,146	FMV	RTV1
				RPeV	RRSV
				FPiV2	
				FNV	
Rby2-45	84,627,146	10.64	586,111	FMV	RTV1
				FPhV	RPiV1
				RPeV	RRSV
				FTV2	NLRV
				FPiV3	RToV
				FTV3	

Table S2. Number of small RNA reads mapped to viruses

Viruses	No. of small RNA reads mapped to	viruses	
	Rby1-21	Rby2-45	6
			7
RPeV	198	3639	
FMV	69	39	8
FPiV2	24		
RTV1	134703	109437	
FNV	43		

RRSV	7108	23558
FPhV		23
FTV2		84
FPiV3		9
FTV3		76
RPiV1		179
RToV		135
NLRV		15

Supplementary Figures

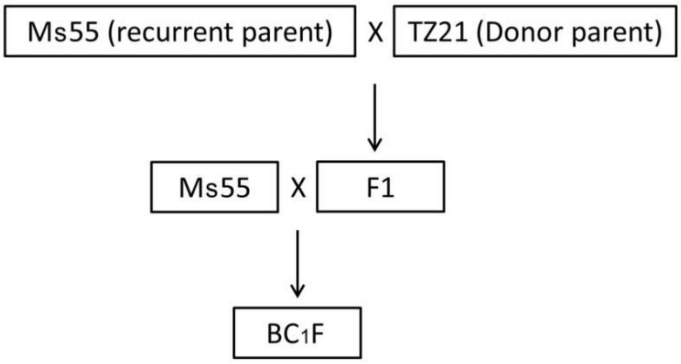

Figure S1. Scheme of backcross breeding used in the current study. Ms55 was used as recurrent 12 parent. TZ21 harboring resistance gene *Bph3* served as donor parent. F1: hybrid first generation; BC_1F: backcross hybrid first generation.

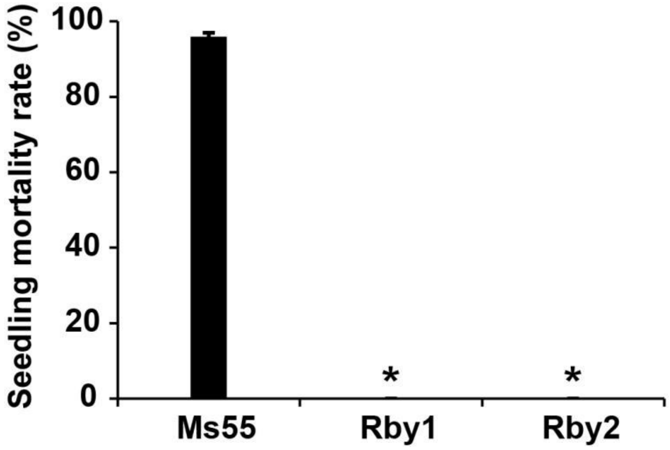

Figure S2. Seedling mortality rate of Ms55, Rby1, and Rby2 infested with BPH. Data were collected 23 19 days post infestation (dpi). About 20 seedlings each.(Error bars, mean of three replicates ±s.e.m. *P < 0.01).

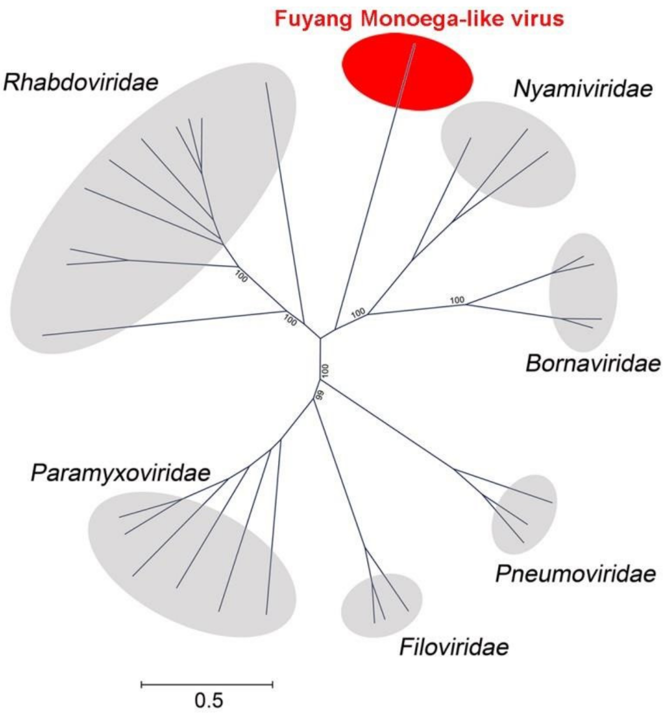

Figure S3. The unrooted ML phylogeny of the novel FMV. Phylogeny of the FMV to viruses within *Mononegavirales* based on RdRp. Each terminal branch represents a single type species representative of a genus.

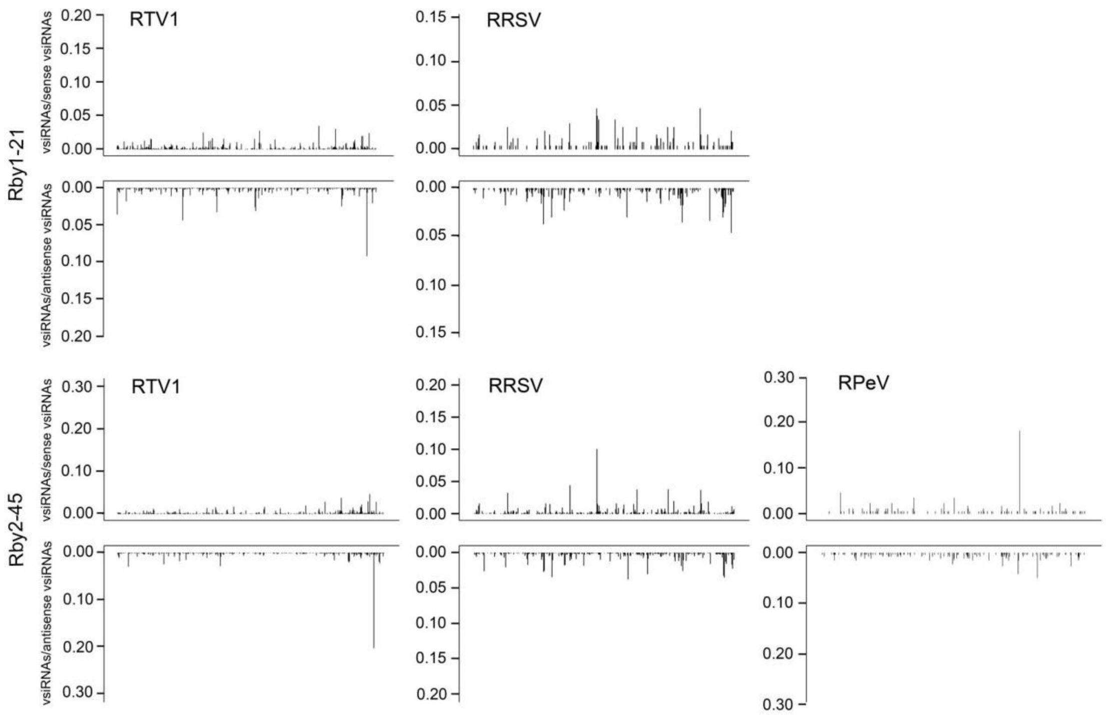

Figure S4. Highly abundance 21-nt vsiRNAs to target the viral genomes. Mapping of the 21 sense and antisense vsiRNAs to the full-length genomes of RTV1 and RRSV in Rby1-21 as well as RTV1,

RRSV and RPeV in Rby2-45. Lines plotted above the *x* axis represent vsiRNAs that map to the positive strand, and those plotted below represent those that mapped to the negative strand.
